# Genetic homogenization of the nuclear ITS loci across two morphologically distinct gentians in their overlapping distributions in the Qinghai-Tibet Plateau

**DOI:** 10.1038/srep34244

**Published:** 2016-09-30

**Authors:** Quanjun Hu, Huichao Peng, Hao Bi, Zhiqiang Lu, Dongshi Wan, Qian Wang, Kangshan Mao

**Affiliations:** 1 MOE Key Laboratory for Bio-resources and Eco-environment, College of Life Science, Sichuan University, Chengdu, 6100065, China; 2 Key Laboratory of Evolution and Adaptation of Plateau Biota, Northwest Institute of Plateau Biology, Chinese Academy of Sciences, Xining, Qinghai 810001, P.R. China; 3 State Key Laboratory of Grassland Ecosystem, School of Life Science, Lanzhou University, 730000 Lanzhou, China; 4 Key Laboratory of Oral Diseases Research, Research Center for Medicine & Biology, Zunyi Medical University, Zunyi 563000, China

## Abstract

Interspecific hybridization and introgression can lead to partial genetic homogenization at certain neutral loci between morphologically distinct species and may obscure the species delimitation using nuclear genes. In this study, we investigated this phenomenon through population genetic survey of two alpine plants (*Gentiana siphonantha* and *G. straminea*) in the Qinghai-Tibet Plateau, where the distributions of two species are partly overlapped. We identified two clusters of chloroplast DNA haplotypes which correspond to the two species, and three clusters of ITS ribotypes. In addition to clusters specific to each species, the third ITS cluster, which was most likely derived from hybridization between the other two clusters and subsequent recombination and concerted evolution, was widely shared by two species in their adjacent areas. In contrast to the morphological distinctiveness of the two species, interspecific gene flow possibly led to genetic homogenization at their ITS loci. The new ITS lineage recovered for species in adjacent areas is distinctly different from original lineages found in allopatric areas. These findings may have general implications for our understanding of cryptic changes at some genetic loci caused by interspecific gene flow in the history, and they indicate that species delimitation should be based on a combination of both nuclear and chloroplast DNA sequence variations.

Hybridization between morphologically distinct species is widespread in plants^1^. It has been especially frequent in the context of climatic changes that caused the distributions of species that were previously geographically isolated to become overlapping^2^,^3^. These hybridization events may have played an important role in triggering plant diversification through homoploid and allopolyploid speciation^4^,^5^,^6^. In addition, interspecific hybridizations can lead to adaptive introgression that increases both phenotype and niche diversity within a species^7^,^8^. Most previous studies have focused on detection of instances of interspecific introgression and identification of cases where the original alleles and haplotypes have been replaced by introgressed forms^8^,^9^,^10^,^11^. However, genetic homogenizations at some nearly neutral loci may be developed under such gene flow and introgressions between species.

To test the latter hypothesis, we collected and compared population genetic data from two gentian species, *Gentiana siphonantha* Maxim. (Fig. 1a) and *G. straminea* Maxim. (Fig. 1b), across their current distributions. Both species now occur in the Qinghai-Tibet Plateau (QTP), where the distributional ranges of all organisms underwent extensive and repeated shrinkage and expansion in response to the Quaternary climatic oscillations^12^,^13^. These two species originated from a common radiation of *G.* sect. *Cruciata* Gaudin. in the QTP before the Pleistocene, but they can be respectively clustered into two well supported clades with the other species of the genus that occur in adjacent regions^14^. The flowers of *G. siphonantha* are arranged in dense, terminal clusters or axillary whorls, with dark-blue corolla and campanulate, and calyx tubes 4–6 mm long whereas *G. straminea* has a thyrose and lax cyme, and a yellow-green and funnelform corolla with calyx tubes 15–28 mm long^15^. *Gentiana siphonantha* occurs in alpine shrubs and meadows in the northeastern QTP (between altitudes of 2200 m and 4500 m) while *G. straminea* is widely distributed in degenerating alpine meadows throughout the QTP (at altitudes ranging from 3000 to 4800 m). The distributions of the two species therefore overlap in the northeastern QTP, with a few populations growing sympatrically (Fig. 1c) and the others occurring parapatrically. Both species are pollinated by bumblebees and in one of their sympatric distributions, the same bumblebee species was found to pollinate both species^16^. Although *G. straminea* flowers earlier than *G. siphonantha*, this prezygotic barrier is incomplete due to a partial overlap in flowering duration, and this is likely to have led to the production of interspecific hybrids and subsequent genetic introgression events^17^.

In the Gentianaceae and other angiosperm plants, chloroplast (cp) DNA is usually inherited maternally through the seeds with low rates of gene flow, while the nuclear ITS (Internal Transcribed Spacer DNA) regions are inherited through both seeds and pollen and exhibit high rates of gene flow^18^. Sequence variations in these two classes of DNA fragments are commonly used to construct plant phylogenies and delimit closely related species in diverse groups^19^,^20^,^21^,^22^,^23^. Introgression of the maternally inherited DNA haplotypes due to low rates of gene flow have been widely detected in regions where the distributions of closely related species overlap^10^,^11^. In addition, hybrid progenies usually display incomplete concerted evolution of the ITS loci, with the allelic sequences being similar to those of both parents^24^,^25^. Further homogenization of parental ITS alleles in the introgressed offspring may lead to the development of a new ITS sequence containing a signature of parent-specific sites^21^. A previous study involving *G. siphonantha* and *G. straminea*, which was based on limited numbers of samples from their allopatric distributions, suggested that they differ from each other with respect to both cpDNA and ITS sequence variations^14^. If introgression events and genetic homogenization do occur in their overlapping distributions, we would expect to find the cpDNA haplotypes specific to one species in the other species and to detect new ITS sequences containing species-specific signatures from both species and/or new mutations. In total, we collected 289 individuals of 32 populations from allopatric and parapatric (or sympatric) distributions of *G. siphonantha* and *G. straminea*. We examined both cpDNA and ITS sequence variations. We aimed to address the following questions: (1) do the cpDNA and ITS sequence variations correspond to morphological delimitations of the two species and (2) if not, can new cryptic hybrid lineages and evidence for genetic homogenization be identified in the regions where their distributions overlap?

## Results

### CpDNA sequence variation within and between the two species

The two chloroplast DNA sequences were combined due to the rarity of recombination events in the chloroplast DNA region, resulting in a total alignment length of 1227 bp to 1252 bp across the 305 individuals studied. Nucleotide substitution had occurred at nine sites, six of which showed transitions (A/G; C/T) and four contained transversions (T/G; C/G), and one indel was present in the *trn*S-*trn*G region (Table 1). In total, these polymorphic sites identified a total of 10 haplotypes (H1-H10) (Table 1).

**Figure 2:**
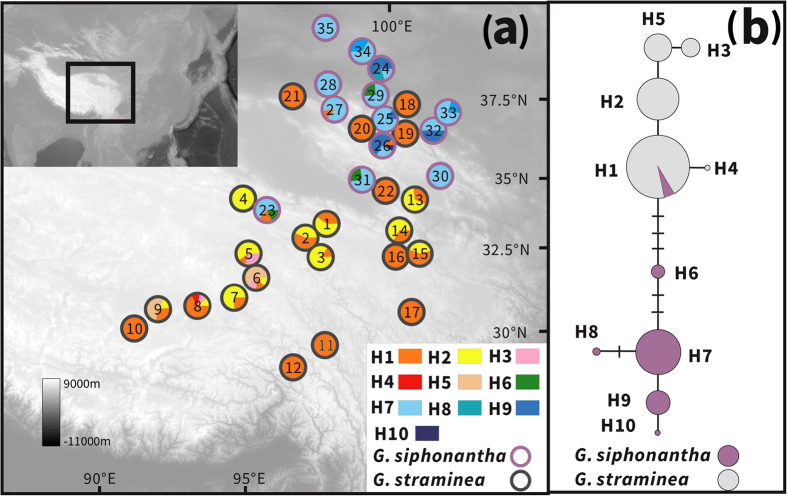
(**a**) Sampling sites and chloroplast haplotype frequencies in the populations of *G. straminea* (black circles) and *G. siphonantha* (white circles) surveyed. (**b**) Network of chloroplast haplotypes. Circle size is proportional to chloroplast haplotype frequency. Map courtesy of ETOPO1, National Geophysical Data Center, NOAA, USA^53^.

Only one haplotype (H1) was shared by the two species, while the others were species-specific (Fig. 2). Haplotype H1 was fixed in three populations (23, 26, 27) of *G. siphonantha* and 10 populations (10–12, 16–22) of *G. straminea* (Table S1, Fig. 2). In *G. straminea*, the next most abundant haplotype was H2, occurring in 12 populations (1–9; 13–15). Three haplotypes, H3, H4 and H5, occurred less frequently in this species. In *G. siphonantha*, H7 was the most frequent, occurring in all populations, while the other haplotypes were distributed sparsely across the other populations. Phylogenetic and network analyses recovered two major clades, one comprising H1-H5, and the other including the five remaining haplotypes (H6-H10) (Fig. 2). With the exception of the shared haplotype H1, this grouping was highly consistent with the morphological distinctness of the two species (Fig. 2).

Molecular variance analysis (AMOVA) of chloroplast sequence diversity for each species attributed half of the variation (50.26%) to between-population differences, and the remainder (49.74%) to within-population effects for *G. straminea*, while for *G. siphonantha,* 75.28% of the total variation was at the within-populations level with only 24.78% attributable to between-population differences (Table S2). Finally, when the two species were pooled together, most of the variation (77.13%) was at the between-species level (*F*_ST_ = 0.86, *P* < 0.01), revealing clear cpDNA divergence between the two species.

Estimates of gene diversity (*H*d) and nucleotide diversity (π) for each population were calculated based on the frequencies of the chloroplast haplotypes (Tables S1 and S3). The total genetic diversity *H*_T_ (0.474; 0.546) across all populations for both *G. siphonantha* and *G. straminea* was clearly higher than the average within-population diversity *H*s (0.356; 0.264). *N*_ST_ (0.209; 0.532) was not significantly higher than *G*_ST_ (0.248; 0.518; P > 0.05) for the two species individually, while a significantly higher value for *N*_ST_ (0.795) than for *G*_ST_ (0.601; P < 0.05) was detected when the two species were analyzed together (Table S3). Mantel tests for each species indicated no significant correlation between geographical and genetic distance (*G. siphonantha*: *r*^*2*^ = 0.001, *P* =  0.354; *G. straminea*: *r*^*2*^ = 0.000, *P* = 0.346; both species: *r*^*2*^ = 0.003, *P* = 0.133), thus suggesting that there was no isolation by distance in the populations studied.

### ITS sequence variation within and between the two species

**Figure 3:**
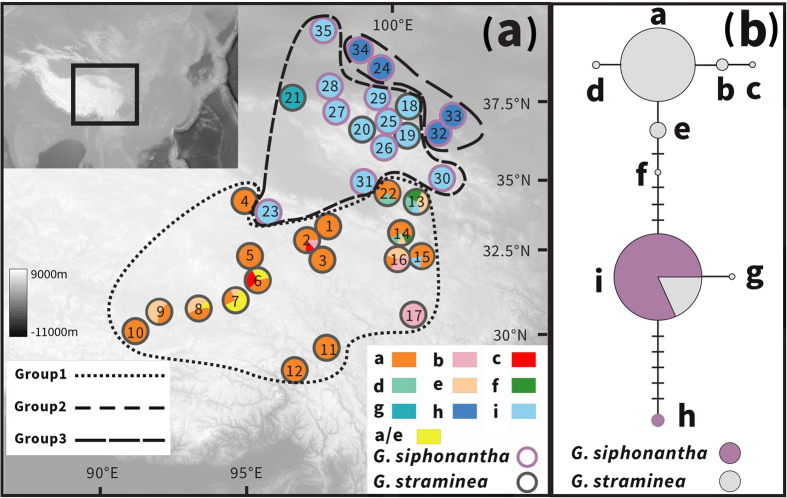
(**a**) Sampling sites and ITS ribotype frequencies in the populations of *G. straminea* (black circles) and *G. siphonantha* (white circles) surveyed. (**b**) Network of ITS ribotypes. Circle size is proportional to ITS ribotype frequency. Based on geographical distribution, cpDNA haplotypes and ITS ribotypes, we divided the sampled location into three groups: group 1 and group 3 as allopatric populations, group 2 as parapatric/sympatric populations. Map courtesy of ETOPO1, National Geophysical Data Center, NOAA, USA^53^.

Most of the sampled individuals showed homozygous ITS sequences according to direct sequencing of the PCR amplified fragments. Only five individuals of population 7 are heterozygous at one site (site 537, C/T). In addition, sequences for up to 20 ITS clones for each individual collected from parapatric or sympatric distributions confirmed the direct sequencing results. The aligned ITS sequence dataset is 627 bp in length with nine sites exhibiting nucleotide substitutions and one indel 3 bp in length (Table 2), and eight genotypes were identified based on these polymorphisms. Seven populations of *G. straminea* contained only the most common ITS type (ITS-a), and this ITS sequence also occurred in another nine populations of the species at a low frequency. Three other ITS ribotypes (ITS-e, ITS-d and ITS-f) co-occurred in two populations (13 and 14). ITS-b occurred in three populations (2, 16 and 17) while ITS-c occurred in two populations (2 and 6). ITS-g was found only in one population (21). In *G. siphonantha*, four populations (24 and 32–34) contained only the ITS-h. However, the other nine populations (23, 25–31 and 35) of this species were fixed for the ribotype ITS-i. This type was also found in parapatrically distributed populations (18–20) of *G. straminea* (Fig. 3).

**Figure 4:**
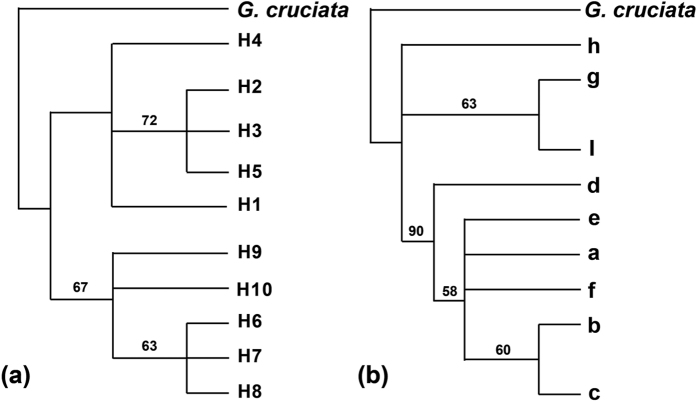
Phylogenetic relationships of chloroplast haplotypes (left: ML tree) and ITS ribotypes (right: MP tree) using *G. cruciata* as the outgroup.

TCS and phylogenetic analyses of the nine ITS ribotypes resolved three distinct lineages (Fig. 4): one containing ITS-a, ITS-b, ITS-c, ITS-d, ITS-e and ITS-f, the second comprising only ITS-h and the third consisting of ITS-i and ITS-g. The first lineage was characterized by the indel that found only in *G. straminea* and the second lineages were specific to *G. siphonantha*. In addition, the third lineage was shared. Most genetic variation based on ITS sequences was found at the among-populations level, for both species. Around 42% of the total genetic variation resided between the two species (*F*_ST_ = 0.80, *P* < 0.01) (Table S2).

### Introgression and gene flow between the two species

We tested introgression using a five-taxon *D-*statistics test, *D*_FOIL_. The *D*_FOIL_ signature is (+, +, 0, 0) for (*D*_FO_, *D*_IL_, *D*_FI_, *D*_OL_) in combination (f, g, i, h), (i, g, i, h) and all five significant cpDNA combinations. This signature means that there exists an introgression between P3 and the ancestral population of P1 and P2 (Table 3).

We further detected the magnitude and direction of gene flow between the two species (Table S4) based on pooled data of cpDNA and ITS regions in MIGRATE, an approach that integrates coalescent theory and maximum likelihood estimation. The result suggests that gene flow between the two species at both cpDNA and ITS regions are asymmetrical. The estimated mean effective number of migrants for ITS (4*N*_e_*m*) is higher from *G. siphonantha* to *G. straminea* (0.62) than the opposite direction (0.24). However, the estimated mean effective number of migrants for cpDNA (2*N*_e_*m*) is *lower* from *G. siphonantha* to *G. straminea* (0.19) than the opposite direction (0.53).

### Ecological niche models and glacial expansion

**Figure 5:**
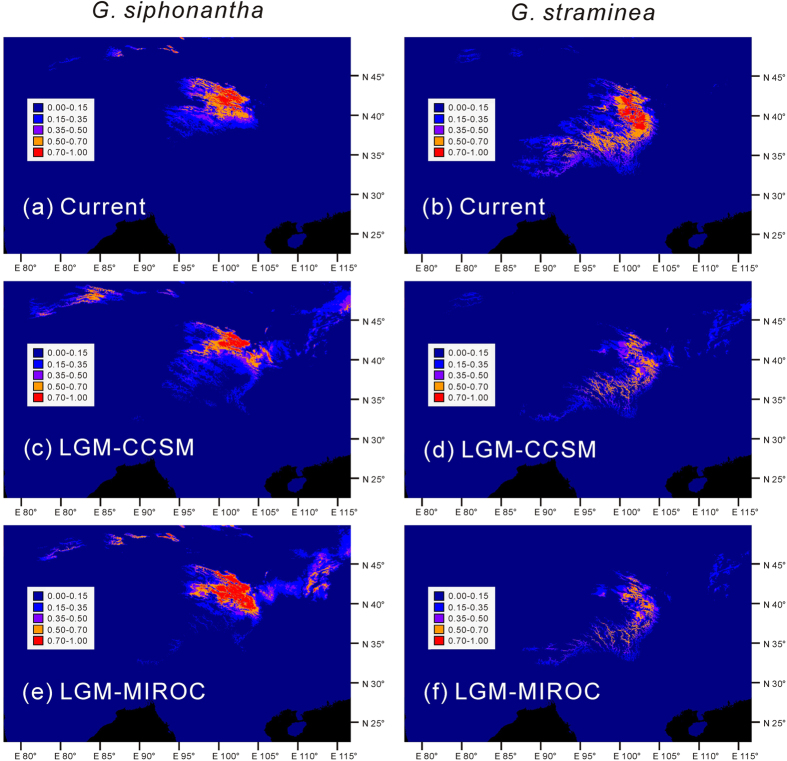
Predicted distribution of *G. siphonantha* and *G. Straminea* based on the ecological niche modeling using MAXENT 3.2. The white dots represent locations used to reconstruct the ecological niche models. The white dots indicate all the used locations in this simulation. Top: The present predicted distribution; Middle: Predicted distribution during the Last Glacial Maximum (LGM) based on the Community Climate System Model version 3 (CCSM) climate simulation; Down: Predicted distribution during the LGM based on the Model for Interdisciplinary Research on Climate version 3.2 (MIROC) climate simulation.

According to the average over the 20 replicates of the MAXENT runs, the areas under the ROC curve (AUC) values for *G. straminea* are 0.972 ± 0.005, 0.974 ± 0.005 and 0.973 ± 0.007 in the present-day model, the LGM-MIROC model and the LGM-CCSM model, respectively; and the AUC values for *G. siphonantha* in these models are, 0.980 ± 0.008, 0.982 ± 0.007 and 0.983 ± 0.007, respectively. All AUC values are very close to 1.0, indicating nearly perfect model performance. As the present-day, the LGM-MIRCO and LGM-CCSM niche models have predicted (Fig. 5), the potential core distribution (habitat suitability index > 0.5) of the two species overlapped in all three models, indicating that they most likely also co-occurred during the LGM. Meanwhile, the potential distribution of *G. straminea* was highly fragmented and this species may have experienced rang-wide population expansion since LGM to achieve the present-day distribution; however, the potential distribution of *G. siphonantha* during LGM is very similar to the present-day, indicating a relatively stable distribution range since LGM.

## Discussion

### Shared polymorphisms between species

Our phylogenetic analyses of chloroplast DNA haplotypes from *G. siphonantha* and *G. straminea* recovered two clades; haplotypes were mostly species-specific with the exception of one widely distributed haplotype belonging to the *G. straminea* clade that was occasionally shared by *G. siphonantha* in the areas where the distributions of both species overlapped. However, phylogenetic analyses of ITS sequences recovered from the two species identified three clades: two specific to each species, while the third clade appeared to be the result of hybridization between the former two clades and subsequent concerted evolution, corresponded to populations in regions where the distributions of the two species overlapped.

To test whether the observed pattern is caused by incomplete lineage sorting or hybridization, we did a *D*_FOIL_ test using populations in group 1 (P1) and group 2 (P2) of *G. straminea*, populations in group 2 (P3) and group 3 (P4) of *G. siphonantha*, and *G. cruciata* was assigned as outgroup (O). The *D*_FOIL_ signature of both cpDNA and ITS indicate the ancestral population of P1 and P2 may have experienced introgression with P3 (Table 3). Meanwhile, MIGRATE suggests that gene flow between both species are asymmetrical (Table S4), higher gene flow occurred at ITS loci from *G. siphonantha* to *G. straminea*, whereas higher gene flow occurred at cpDNA loci from the latter to the former species. These findings suggested the occurrence of cpDNA introgressions, extensive genetic homogenization at ITS loci under interspecific gene flow, and the development of a new ITS lineage in the overlapping distributions of the two species.

### Homogenization of the ITS loci

Interspecific hybridization events can result in the retention of parental alleles with additive sites or double peaks in chromatograms of nuclear ITS sequences that differentiate the two parents in F_1_ hybrids^21^,^24^. Sexual reproduction in subsequent generations of hybrids or backcrosses may lead to the recombination of parent alleles, and after complex interplays among hybridization and concerted evolution, a single but mosaic ITS sequence that comprise the species-specific mutation sites of both parental species might be generated^26^. In this study, we found three groups of ITS sequences, and their occurrences correspond to the allopatric distributions of *G. siphonantha* and *G. straminea*, and their overlapping distributions, respectively (Fig. 3).

It has been suggested that ITS variations are species-specific, and can therefore be used for species delimitation and identification^23^. However, it is interesting that in our study, the third lineage (including both ITS-i and ITS-g, Figs 3 and 4) contained three nucleotide mutations specific to the allopatric *G. straminea* populations (sites 195: G, 549: C and 561: G) (Table 2) and two specific to the allopatric *G. siphonantha* populations (sites 116: C and 184: T) (Table 2). In addition, we found that a mutation at site 228 (T), which appeared in this lineage, seemed to be absent from most allopatric populations of the two species. Our analyses thus suggested that the third lineage might have derived from a hybrid combination of the other two lineages (Table 2) that characterize the two species in their allopatric distributions, with the further occurrence of a lineage-specific mutation.

Genetic homogenization of ITS sequences in the region of overlap between *G. siphonantha* and *G. straminea* suggests that gene flow between these species has been frequent and continued over a long period of time in order to cause the same mutation to be present in both species. In their current distributions, both species have the same pollinators, but they are to some extent ecologically isolated by flowering time^16^. *Gentiana straminea* flowers earlier than *G. siphonantha*, but their flowering times partly overlap, and this has probably led to the frequently observed hybrids in the field^17^. Interspecific crossing experiments suggested incomplete post-zygotic reproductive isolation, because interspecific hybridization usually produced a few fertile and viable seeds^16^. Most examples of pre-zygotic isolation of closely related species, especially as a result of different flowering times, may have resulted from the reinforcement of reproductive isolation in order to avoid the unnecessary costs of producing interspecific hybrids in co-occurring distributions^27^. Although this hypothesis needs further confirmation in the case of *G. siphonantha* and *G. straminea*, it is likely that the flowering times of the two species overlapped more in the past when the species first made contact than they do at present. Gene flow between them in the overlapping distributions may therefore have been more extensive in the past than the present.

At the same time, ecological niche modeling suggests that highly suitable niche for both species overlapped in the northeastern QTP in the past (LGM), confirming that the two species not only co-occur at present, but also co-occurred in the past. Thus, a continuation of gene flow from the past to the present day may have caused the concerted evolution of ITS loci and led to the homogenization of ITS sequences in the overlapping regions of the two species as observed in this study.

### CpDNA introgressions between species

In this study, two groups of cpDNA haplotypes (Figs 2 and 4) were recovered across the two species. One group, consisting of haplotypes H6 to H10, was found only in *G. siphonantha*. The other, comprising H1 to H5 (Fig. 2) was found mainly in *G. straminea*. This group was characterized by a long indel. However, the commonest haplotype H1 in this group was also found in three populations of *G. siphonantha* (populations 23, 26, 27; Fig. 2). This shared haplotype may have originated from introgression between the two species or through retention of ancestral polymorphisms because of incomplete lineage sorting^28^. However, introgression seems the more likely interpretation for three reasons. First, this shared haplotype is distributed geographically in parapatry, while haplotypes that are shared due to incomplete lineage sorting occur in both allopatric and parapatric regions^29^,^30^,^31^. Second, the two distinct groups differ from each other by at least four mutational steps, much more than the difference between haplotypes within each lineage. This contrasts with haplotypes shared because of incomplete lineage sorting. Third, the *D*_FOIL_ signature rules out the possibility of incomplete lineage sorting and supports a hypothesis where introgressions occurred in the past (Table 3). At last, homogenization at the ITS loci in the overlapping distribution of the two species reflect frequent historical interspecific genetic exchanges in this area, which also lend support to the introgression cpDNA variation. Therefore, we suggest that it is hybridization and subsequent introgression that have caused the sharing of one chloroplast haplotype identified here.

Apart from the shared haplotype, we found that all of the five haplotypes recovered for *G. straminea* occurred in the central platform of the plateau (in populations 5–9), from where genetic diversity decreased toward the edge. This distributional pattern suggests that glacial refugia may have been located near the platform of the plateau and that postglacial expansions took place mainly toward the northerly edge of the plateau^32^. All populations in the north were dominated by a single haplotype (H1) during this postglacial expansion. However, four haplotypes recovered for *G. siphonantha* were sparsely distributed in the overlapping regions of the two species in the northeastern QTP (populations 26 and 31) or in the allopatric regions (for example, populations 24 and 34). The haplotype distributions of *G. siphonantha* populations suggested that multiple refugia may have been occupied by this species and that the current distributions were derived from these refugia; this is consistent with ecological niche modeling, which reveals highly similar distribution of this species during LGM and at the present (Fig. 5). In the north of the QTP, *G. straminea* may have invaded, in large numbers, the distributional range already occupied by *G. siphonantha* (Fig. 5). These expansions probably led to interspecific hybridization and gene flow between the two species, followed by recombination and then resulted in genetic homogenization and concerted evolution of ITS regions between the two species and led to the shared chloroplast haplotype.

### Implications

Because cpDNA and ITS sequence variations are used together as markers to identify different species or evolutionary lineages^22^,^23^, four distinct lineages can be recognized across *G. siphonantha* and *G. straminea* if both cpDNA and ITS lineages are considered (Fig. 3A: group 2 could be further divided into two lineages based on cpDNA haplotypes). The development of a new ITS lineage for these species in their overlapping distributions suggests that in these regions the species developed cryptic differentiation from the populations found in allopatric distributions. Since distributional shifts affected numerous species during climatic oscillations in the past, especially in the climate-sensitive QTP^33^, our results highlight the importance of cryptic biodiversity caused by interspecific hybridization events, which has been largely neglected in previous studies. More attention should be paid to interspecific hybridization and its aftermath in the future, especially during explorations of biodiversity. In addition, sequence variations in both the ITS region and the chloroplast DNA should be used together for species delimitation and identification in such studies.

## Material and Methods

### Population sampling

We sampled 22 populations for *G. straminea* and 13 populations for *G. siphonantha* (Table S1, Figs 2 and 3). All sampled individuals had the morphological traits typical of their respective species and could be unambiguously identified as either *G. straminea* or *G. siphonantha*. We took particular care to avoid sampling individuals with intermediate morphologies^17^. Within each population, the sampled individuals were separated by at least 50 meters. In the northeastern QTP, where the two species are distributed together, any two populations sampled from the same site were separated by at least 2 km. A total of 15 populations from the two species (for example, populations 4 and 23, Table S1) were considered to be parapatric or sympatric. Although some specimens had previously been recorded in northern China, all of which were on mountains with altitudes between 2000 m and 3000 m in the Ningxia Autonomous Region, Shanxi, Shannxi, Hebei and Hubei provinces, we have not found any natural distribution in these areas (artificial cultivation was occasionally observed), despite intensive searches from 2003–2005. The sparse records of natural distributions in these areas are at least 30 years old, and both species could have become extinct in the recorded localities, possibly due to climatic changes, or, due to human activity given that the roots of both species have been used as important traditional medicines^15^. The sampled populations cover the current distribution ranges of the two species in the QTP (Figs 2 and 3, Table S1). Fresh leaves in the field were collected, dried and stored in silica gel. Voucher specimens are preserved in the Northwest Plateau Institute of Biology (HNWP), Chinese Academy of Sciences.

### DNA extraction, amplification and sequencing

Total genomic DNA was extracted from the silica dried leaves of each individual following a modification of the CTAB method according to Doyle & Doyle^34^. Two cpDNA fragments (*trn*S-*trn*G and *trn*L-*trn*F) and an ITS region (including ITS1, 5.8S and ITS2) were amplified and sequenced using primers from Hamilton^35^ and White *et al.*^36^. Polymerase chain reaction (PCR) was performed in a 25-μl volume, containing 10–40 ng plant DNA, 50 mM Tris-HCI, 1.5 mM MgCl_2_, 250 μg/mL BSA, 0.5 mM dNTPs, 2 μM of each primer, and 0.75 unit of Taq polymerase. Reactions were conducted using the following program: 4 min at 94 °C, 35 cycles of 50 seconds at 94 °C, 1 min of annealing at 53 °C, 1.25 min at 72 °C, with a final 8 min extension at 72 °C, and all products were held at 4 °C until required for further processing. PCR amplifications were purified using a TIANquick Midi Purification Kit following the manufacturer’s protocol (TIANGEN), and purified PCR products were directly sequenced. We selected one individual from each population where two species occur parapatrically and one allopatric population of *G. straminea* (population 7) for further sequencing of different clones from the same individual (a total of 16 individuals from 16 populations of two species). PCR products were cloned into the pGEM T-vector (Promega, Madison, Wis) and 20 clones were chosen and sequenced using the T7 promoter and M13-21mer primers.

We further amplified and sequenced the corresponding DNA fragments from one individual of *G. cruciata* from Europe as an outgroup for phylogenetic analyses. All sequences obtained were aligned using the default parameters of CLUSTAL X^37^ and checked manually. Sequence boundarie**s** were defined by comparison to the published sequences from *G. straminea* downloaded from GenBank. All sequences are available from GenBank, accession numbers HM598120-HM598122(for *trn*L-F sequences), DQ398737, DQ497591, DQ497586, HM566108-HM566110 (for *trn*S-G sequences) and HM598091-HM598098, DQ497573, DQ497574, DQ398630-DQ398631(for ITS sequences).

### Phylogenetic constructions and networks

Different sequences identified within the cpDNA and ITS datasets were numbered as cpDNA haplotypes and ITS types respectively. Most ITS types were obtained using a direct sequencing approach. Our cloning analysis suggested that all ITS types in the individuals sampled from the overlapping distributions of the two species have undergone completely concerted evolution and are therefore population-specific; however, one ITS type with a single additional nucleotide at one site indicating possible hybridization was detected in population 7. There are multiple copies of ITS in plant genome and these DNA regions usually subject to concerted evolution, so genetic variation among copies within each individual could indicate naturally occurring species**-**specific (or population**-**specific) variation among copies and a failure of concerted evolution; alternatively, it might be the result of more recent hybridization and reticulation.

Both cpDNA haplotypes and ITS types were subjected to network analysis using TCS version 1.21 according to the acceptance criteria given by Clement *et al.*^38^. All indels were coded as additional single binary characters. These two datasets were also used for phylogenetic reconstructions by running maximum-likelihood analysis with PAUP* 4.0b10 39 and with MrBayes 3.0 40,41. We used MODELTEST^42^ to select parameters and assumptions for the maximum-likelihood analyses. The heuristic search parameters for these analyses were simple addition of sequences of taxa with TBR branch swapping, MULTREES and COLLAPSE on MP analyses (equally weighted characters and nucleotide transformations) involving a heuristic search strategy with 100 replicates of random addition of sequences, in combination with ACCTRAN character optimization, MULPARS+TBR branch swapping and STEEPEST DESCENT options on. For Bayesian analyses, four chains were used per run (three heated and one cold), and each analysis was repeated three times: twice for two million generations, with the final analysis running for 10 million generations. We used both bootstrap values and Bayesian posterior probabilities to assess branch support. Bootstrap analysis was calculated from 1000 replicates using a heuristic search with simple addition, with TBR and MULPARS options on, in PAUP* 4.0b10.

### Genetic diversity and phylogeographic analyses

Average gene diversity within populations (*H*_S_), total gene diversity (*H*_T_), and estimates of the population differentiation statistics *G*_ST_ and *N*_ST_, based on the chloroplast dataset, were calculated using the program PERMUT with 1000 permutation tests^43^. *G*_*ST*_considers only the frequencies of haplotypes or ITS types, while *N*_ST_ takes into account both haplotype frequencies and their genetic distances. The statistical significance of phylogeographic structure was determined by testing whether the value of *N*_ST_ was significantly larger than the value of *G*_ST_ using a permutation test with 1000 random permutations of haplotypes across populations. Genetic variation within populations and among populations within each species and between the two species, as well as estimates of unbiased haplotype diversity (*H*_E_) and nucleotide diversity (π), were generated by analyses of molecular variance^44^ using the program Arlequin version 3.0^45^. Measures of DNA divergence between populations and species (*F*_ST_) were calculated, and their significance was tested using 10000 permutations. The significance of isolation by distance between populations was tested using Mantel tests with 1000 random permutations on matrices of pairwise population *F*_ST_ values and the natural logarithms of geographical distances^46^ for the two species together and separately. Pairwise *F*_ST_ values between populations were estimated using Arlequin while geographical distances between populations were calculated with the aid of the program available at www.indo.com/distance/.

### Detection of introgression and gene flow analysis

To test whether introgression or incomplete lineage sorting cause the sharing of cpDNA haplotype and nuclear ITS ribotype between the two species, we conducted five-taxa *D*_FOIL_ tests (including four separate components: *D*_FO_, *D*_IL_, *D*_FI_ and *D*_OL_) for each combination of haplotypes or ribotypes in P1, P2, P3, P4 and O using python script from http://www.github.com/jbpease/dfoil and set *p*-value to 0.05 for significance. Different combination of positive and negative values of *D*_FO_, *D*_IL_, *D*_FI_ and *D*_OL_ will not only be able to differ introgression from incomplete lineage sorting, but also the direction and significance of introgression that occurred between different groups^47^.

Meanwhile, we estimated the scaled mutation rates, θ, of the two species (4*N*_*e*_μ, where *N*_*e *_= effective population size and μ = mutation rate) and the effective number of migrants (2*N*_*e*_m, where *N*_*e*_ is the effective population size and m is the migration rate) per generation using the software package MIGRATE version 3.2.6. We adopted the mutation rate of 2 × 10^−9^ and 1.9 × 10^−8^ per gamete per generation for cpDNA and ITS respectively according to previous studies^14^,^48^. We conducted these estimations based on 20 short chains (10,000 trees) and three long chains (1,000,000) with 10,000 trees discarded as the initial ‘burn-in’.

### Ecological niche modeling

We used ecological niche models (ENMs) based on the maximum entropy method implemented in MAXENT 3.3.3k^49^ to predict the distributional ranges of *G. siphonantha* and *G. straminea* at present and during the LGM (0.021-0.018 Mya). Nineteen environmental variables and altitude all over the world during LGM (MIROC^50^; CCSM^51^) and at present were retrieved from the WorldClim database (http://www.worldclim.com) with a resolution of 2.5 arc-min. To avoid strong collinearity of environmental variables which could lead to model over-fitting, Pearson correlation for bioclimatic variables was conducted and the less correlated variables were retained (Pearson correlation value, <0.8). In total, altitude and 8 bioclimatic variables (Mean Diurnal Range, Isothermality, Mean Temperature of Warmest Quarter, Temperature Annual Range, Precipitation of Wettest Quarter, Precipitation of Driest Quarter, Precipitation of Seasonality, and Precipitation of Warmest Quarter) were used to model the distribution of the two species.

Ecological niche modeling was constructed according to current environmental factors and then projected for the LGM. In total, 65 and 75 presence sites for *G. siphonantha* and *G. straminea*, respectively, were employed to train the model. Among these, 13 and 23 precise locations, where leaf samples were taken (Table 1), were measured using an Etrex handheld GPS unit (Garmin, Olathe, KS, USA); the remaining locations (Table S1) were achieved from herbarium record at the Chinese Virtual Herbarium (http://www.cvh.ac.cn/). We employed 20 replicates by using 80% of the distribution coordinates for training and 20% for testing, and the model with the best AUC value was adopted. We considered that an AUC score greater than 0.7 indicates a satisfactory model performance^52^. At the same time, the “10 percentile presence” threshold approach was employed because presence-only data were available here. Graphics for each predicted species distribution model were drawn using DIVA–GIS 7.5.

## Additional Information

**How to cite this article**: Hu, Q. *et al.* Genetic homogenization of the nuclear ITS loci across two morphologically distinct gentians in their overlapping distributions in the Qinghai-Tibet Plateau. *Sci. Rep.* **6**, 34244; doi: 10.1038/srep34244 (2016).

## Supplementary Material

Supplementary Information

## Figures and Tables

**Figure 1:**
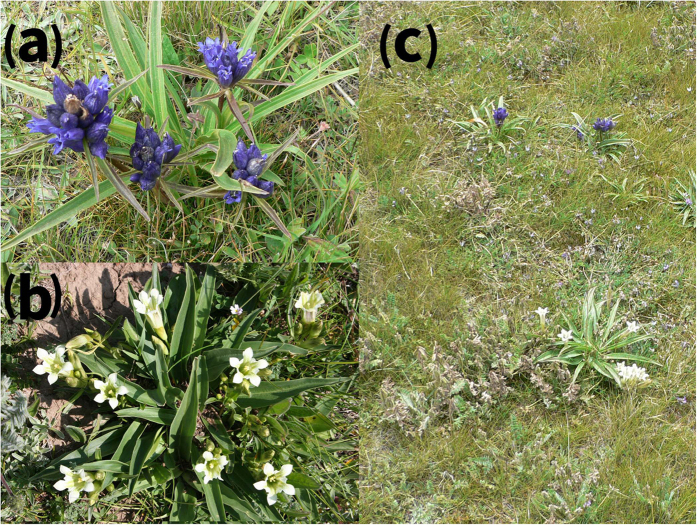
(**a**) *Gentiana siphonantha* flowers are arranged in dense, terminal clusters or axillary whorls, with dark-blue corolla and campanulate, and calyx tubes 4–6 mm long. (**b**) *Gentiana straminea* has a thyrose and lax cyme, and a yellow-green and funnelform corolla with calyx tubes 15–28 mm long. (**c**) The sympatric occurrence of two species in the northeastern QTP. Photos courtesy of Prof. Jianquan Liu.

**Table 1:** Variable sites in cpDNA fragments in the ten chlorotypes of *G. straminea* and *G. siphonantha*.

Chlorotype	*trn*S-*trn*G							*trn*L-*trn*F		
	41	278	444	452	473	532	546	453	494	676
H1	▲	T	C	G	G	C	A	A	C	G
H2	▲	G	C	G	G	C	A	A	C	G
H3	▲	G	C	G	A	C	A	A	C	G
H4	▲	T	C	G	G	T	A	A	C	G
H5	▲	G	C	G	G	C	G	A	C	G
H6	■	T	A	T	G	C	A	A	C	G
H7	—	T	A	T	G	C	A	A	C	G
H8	—	T	A	T	G	C	A	A	T	A
H9	—	T	A	G	G	C	A	A	C	G
H10	—	T	A	G	G	C	A	G	C	G

▲ = TGGATATGTACACATAGATATTAT; ■ = TATATAGATATTAT.

**Table 2:** Nucleotide site variations in ITS sequences found in *G. straminea* and *G. siphonantha*.

Population	Nucleotide sites in ITS sequence										ITS-type
	42	116	184	188	195	226	228	537	549	561	
*G. straminea*											
Pop1, 3–6, 8–12, 15, 22	A	▲	G	G	G	A	G	T	C	G	a
Pop2	A	▲	G	G	G	A	G	T	C	G	a
Pop2	A	▲	G	T	G	A	G	T	C	G	b
Pop7	A	▲	G	G	G	A	G	T	C	G	a
Pop7*	A	▲	G	G	G	A	G	Y	C	G	a/e
Pop13	A	▲	G	G	G	A	G	C	C	G	e
Pop13	A	▲	G	G	G	T	T	C	C	G	f
Pop13	A	▲	T	G	G	A	G	T	C	G	d
Pop14	A	▲	G	G	G	A	G	C	C	G	e
Pop14	A	▲	G	G	G	A	G	T	C	G	a
Pop14	A	▲	G	G	G	T	T	C	C	G	f
Pop14	A	▲	T	G	G	A	G	T	C	G	d
Pop16	A	▲	G	G	G	A	G	T	C	G	a
Pop16	A	▲	G	T	G	A	G	T	C	G	b
Pop16	A	▲	G	T	G	A	G	T	T	G	c
Pop17	A	▲	G	T	G	A	G	T	C	G	b
Pop18–20	C	C	T	G	G	T	T	T	C	G	i
Pop21	A	C	T	G	G	T	T	T	C	G	g
*G. siphonantha*											
Pop23, 25–31, 35	C	C	T	G	G	T	T	T	C	G	i
Pop24, 32–34	A	C	T	G	A	A	G	T	T	T	h

Y = T/C; ^*^indicate ITS sequences with additional nucleotides; ▲ = TATG.

**Table 3:** D_FOIL_ statistics for *G. straminea* and *G. siphonantha*.

Data	P1	P2	P3	P4	O								
	*G. straminea*		*G. siphonantha*			*D*_FO_		*D*_IL_		*D*_FI_		*D*_OL_	
	Group1	Group2	Group2	Group3	*G.cruciata*	*D*	*P*-value	*D*	*P*-value	*D*	*P*-value	*D*	*P*-value
ITS	f	g	i	h	—	1	0.02535*	1	0.02535*	−1	0.0833	−1	0.0833
	b	g	i	h	—	0.2	0.6547	1	0.02535*	−1	0.02535*	−0.2	0.6547
	a	g	i	h	—	0.2	0.6547	1	0.02535*	−1	0.0455*	0	1
	i	g	i	h	—	1	0.02535*	1	0.02535*	0	1	0	1
	e	g	i	h	—	0.2	0.6547	1	0.02535*	−1	0.02535*	−0.2	0.6547
	d	g	i	h	—	0.2	0.6547	1	0.02535*	−1	0.0833	0.3	0.5637
	c	g	i	h	—	−0.2	0.6547	1	0.02535*	−1	0.01431*	0	1
cpDNA	H2	H1	H1	H8	—	1	0.0455*	1	0.0455*	−1	1	−1	1
	H4	H1	H1	H8	—	1	0.0455*	1	0.0455*	−1	1	−1	1
	H3	H1	H1	H8	—	1	0.0455*	1	0.0455*	−1	0.1573	−1	0.1573
	H5	H1	H1	H8	—	1	0.0455*	1	0.0455*	−1	0.1573	−1	0.1573
	H1	H1	H1	H8	—	1	0.0455*	1	0.0455*	0	1	0	1

^*^P < 0.05.
