# Supplementary material for: Genetic homogenization of the nuclear ITS loci across two morphologically distinct gentians in their overlapping distributions in the Qinghai-Tibet Plateau
Source: Sci Rep. 2016 Sep 30;6:34244. doi: 10.1038/srep34244 (PMC5043236; doi:10.1038/srep34244)
Supplement: Supplementary Information [file srep34244-s1.pdf]

Genetic homogenization of the nuclear ITS loci across two morphologically distinct gentians in their overlapping distributions in the Qinghai-Tibet Plateau

Quanjun Hu [a,*], Huichao Peng[b,*], Hao Bi [a,*], Zhiqiang Lu [c], Dongshi Wan [c], Qian Wang[a,d], Kangshan Mao [a,**]

[a]MOE Key Laboratory for Bio-resources and Eco-environment, College of Life Science, Sichuan University, Chengdu, 6100065, China;

[b]Key Laboratory of Evolution and Adaptation of Plateau Biota, Northwest Institute of Plateau Biology, Chinese Academy of Sciences, Xining, Qinghai 810001, P.R. China;

[c]State Key Laboratory of Grassland Ecosystem, School of Life Science, Lanzhou University, 730000 Lanzhou, China;

[d]Key Laboratory of Oral Diseases Research, Research Center for Medicine & Biology, Zunyi Medical University, Zunyi 563000, China

* These authors contributed equally to this work

[**] **Corresponding authors:**
Kangshan Mao
Key Laboratory for Bio-resources and Eco-environment, College of Life Science, Sichuan University, E-mail: maokangshan@163.com, maokangshan@scu.edu.cn

Table S1 Sample locations**, chlorotypes in each population, and estimates of gene diversity (H_d) and nucleotide diversity (π) for each population.

Code	Location	Lat.	Long.	Alt. (m)	N	Haplotypes (Frequencies %)	H_d	π
G. straminea								
1	Chenduo, QH	33.40°	97.31°	3670	10	H1(40.0), H2(60.0)	0.53333	0.00096
2	Yushu, QH	33.06°	97.17°	4330	16	H1(56.2), H2(43.8)	0.52500	0.00095
3	Yushu, QH	32.12°	97.04°	3540	7	H1(14.3), H2(85.7)	0.28571	0.00052
4	Qumalai, QH	34.10°	96.15°	4453	14	H2(100.0)	0.00000	0.00000
5	Nangqian, QH	32.23°	96.47°	4041	10	H1(10.0), H2(60.0), H3(30.0)	0.60000	0.00121
6	Dingqing, XZ	31.69°	94.92°	4900	8	H1(12.5), H2(12.5) H3(12.5), H5(62.5)	0.64286	0.00187
7	Suxian, XZ	31.95°	93.79°	4100	8	H2(75.0), H5(25.0)	0.42857	0.00077
8	Naqu, XZ	31.75°	92.72°	4280	9	H1(66.7), H2(11.1) H3(11.1), H4(11.1)	0.58333	0.00151
9	Naqu, XZ	31.77°	92.58°	4700	7	H1(28.6), H2(14.3), H5(57.1)	0.66667	0.00155
10	Dangxiong, XZ	30.60°	91.50°	4669	8	H1(100.0)	0.00000	0.00000
11	Bangda, XZ	30.46°	97.17°	4370	8	H1(100.0)	0.00000	0.00000
12	Bomi, XZ	29.65°	96.20°	3160	7	H1(100.0)	0.00000	0.00000
13	Maqin, QH	34.63°	100.23°	3580	9	H1(33.3), H2(66.7)	0.50000	0.00090
14	Dari, QH	33.30°	100.42°	4020	9	H1(33.3), H2(66.7)	0.50000	0.00090
15	Makehe, QH	32.65°	100.93°	3540	10	H1(60.0), H2(40.0)	0.53333	0.00096
16	Seda, SC	32.30°	100.35°	3920	5	H1(100.0)	0.00000	0.00000
17	Daofu, SC	30.82°	101.27°	3510	5	H1(100.0)	0.00000	0.00000
18	Gangca, QH	37.06°	100.71°	3476	8	H1(100.0)	0.00000	0.00000
19	Riyueshan, QH	36.43°	101.08°	3440	9	H1(100.0)	0.00000	0.00000
20	Jiangxigou, QH	36.65°	99.97°	3225	9	H1(100.0)	0.00000	0.00000
21	Delingha, QH	37.45°	97.33°	3502	9	H1(100.0)	0.00000	0.00000
22	Huashixia, QH	35.42°	97.31°	4313	8	H1(100.0)	0.00000	0.00000
G. siphonantha								
23	Qumalai, QH	34.10°	96.15°	4453	22	H1(18.2), H6(18.2), H7(63.6)	0.55411	0.00052
24	Haibei, QH	38.14°	100.52°	3500	10	H7(10.0), H8(20.0), H9(70.0)	0.51111	0.00097
25	Huangyuan, QH	36.72°	101.22°	2682	7	H7(85.7), H9(14.3)	0.28571	0.00024
26	Riyueshan, QH	36.43°	101.08°	3447	12	H1(8.3), H7(25.0) H9(58.4), H10(8.3)	0.63636	0.00061
27	Tianjun, QH	37.10°	98.87°	3570	8	H1(12.5), H7(87.5)	0.25000	0.00041
28	Delingha, QH	37.45°	97.33°	3504	9	H7(100.0)	0.00000	0.00000
29	Tianjun, QH	37.15°	99.88°	3193	8	H6(25.0),H7(75.0)	0.42857	0.00035
30	Zeku, QH	35.05°	101.08°	3820	6	H7(100.0)	0.00000	0.00000
31	Huashixia, QH	35.42°	97.31°	4313	8	H6(25.0),H7(75.0)	0.42857	0.00035
32	Xining, QH	36.62°	101.78°	2250	6	H7(50.0),H9(50.0)	0.60000	0.00050
33	Tianzhu, GS	37.44°	102.60°	3128	5	H7(80.0),H9(20.0)	0.4	0.00033
34	Sunan，GS	38.86°	99.62°	3270	6	H7(66.7),H9(33.3)	0.533	0.00044
35	Yumen，GS	39.83°	97.66°	2344	5	H7(100.0)	0	0

Abbreviations: QH, Qinghai; XZ, Xizang; SC, Sichuan; GS, Gansu

Table S2 Analysis of molecular variance (AMOVA) of cpDNA and ITS variation in *G. straminea* and *G. siphonantha*

Source of variation	d.f.	SS	VC	PV (%)	Fixation index
**Chloroplast DNA sequence variation**					
G. straminea* and *G. siphonantha					
Between *G. straminea* and *G. siphonantha*	1	174.908	1.22246Va	77.13	F_{sc} = 0.37705**
Among populations within **the** two species	33	46.38	0.13669Vb	8.62	F_{st} = 0.85752**
Within populations	270	60.975	0.22583Vc	14.25	F_{ct} = 0.77128**
Total	304	282.262	1.58498		
G. straminea					
Among populations	21	28.617	0.14069 Va	50.26	F_{st} = 0.50261**
Within populations	170	23.67	0.13923Vb	49.74	
Total	191	52.286	0.27993		
G. siphonantha					
Among populations	12	9.132	0.06630 Va	24.72	F_{st} = 0.24724**
Within populations	99	19.984	0.20185 Vb	75.28	
Total	111	29.116	0.26815		
**ITS sequence variation**					
G. straminea* and *G. siphonantha					
Between *G. straminea* and *G. siphonantha*	1	23.339	0.17298Va	37.99	*Fsc*=0.67795**
Among populations within **the** two species	33	49.388	0.19143Vb	42.04	*Fst*=0.80029**
Within populations	226	20.552	0.09094Vc	19.97	*Fct*=0.37988**
Total	260	93.28	0.45535		
G. straminea					
Among populations	21	31.091	0.18450Va	55.33	*Fst*=0.55334**
Within populations	138	20.552	0.14893Vb	44.67	
Total	159	51.644	0.33343		
G. siphonantha					
Among populations	12	18.297	0.20161Va	100	*Fst* =1.00000**
Within populations	88	0.000	0.00000 Vb	0.00	
Total	100	18.297	0.20161		

Abbreviations: d.f., degrees of freedom; SS, sum of squares; VC, variance component; PV, percentage of variation; F_{SC}, correlation within populations relative to groups; F_{ST}, correlation within populations relative to total; F_{CT}, correlation of chlorotypes within groups relative to total. ** represents P < 0.01, 1000 permutations

Table S3 Genetic diversity estimates for cpDNA in *G. straminea* and *G. siphonantha*

	H_S	H_T	G_{ST}	N_{ST}
G. straminea	0.264 (0.0596)	0.546 (0.0677)	0.518 (0.0774)	0.532 (0.0659)
G. siphonantha	0.356 (0.0641)	0.474 (0.0863)	0.248 (0.0774)	0.209 (0.0237)
**Total**	0.294 (0.0444)	0.746 (0.0334)	0.601 (0.0487)	0.795 (0.0289)

N_{ST} is not significantly larger ($P>0.05$) than G_{ST} in either *G. straminea* or *G. siphonantha*, while a significantly larger value ($P<0.05$) for N_{ST} than for G_{ST} is detected in 'Total'.

Table S4 Effective population size in G*. straminea and G. siphonantha* and effective migration rates (*N_e*m) between species, estimated by coalescent theory and a maximum-likelihood-based approach based on plastid dataset (symmetric gene flow, asymmetric Theta).

	**ITS**		**cpDNA**	
	G. straminea	***G. siphonantha***	***G. straminea***	***G. siphonantha***
**θ**	0.00239	0.00048	0.00058	0.00097
	0.00007-0.00467	0-0.00213	0-0.00227	0-0.00273
M_{12}		506.8		545.1
		73.3-990.0		131.3-994.7
M_{21}	258.2		322.1	
	0-796.0		0-842.7	
N_e	10500	2110	96700	162000
	307-20500	0-9340	0-378000	0-455000
Migrant Number $_{12}$		0.24*		0.53**
		0-2.11		0-2.72
Migrant Number $_{21}$	0.62*		0.19**	
	0-3.72		0-1.91	

M_{12}: Migration rate from *G. straminea* to *G. siphonanth*
M_{21}: Migration rate from *G. siphonanth* to *G. straminea*
* The effective number of migrants ($4N_em$) from *G. straminea* to *G. siphonanth* per generation (3 years).
** The effective number of migrants ($2N_em$) from *G. straminea* to *G. siphonanth* per generation (3 years).
The range estimates given below each value are the 95% confidence limits.

Table S5. Sampling locations of *Gentiana siphonantha* and *G. straminea* for the inference their potential distribution based on ecological niche modelling in MAXENT. Population numbers of these populations that were subjected to genetic survey are identical to Table S1, and their assignments of ITS lineage are the same as presented in Fig. 3.

Species	Longtitude	Latitude	Population Number	ITS lineage
G. straminea	97.31	33.40	1	Group 1
G. straminea	97.17	33.06	2	Group 1
G. straminea	97.04	32.12	3	Group 1
G. straminea	96.15	34.10	4	Group 1
G. straminea	96.47	32.23	5	Group 1
G. straminea	94.92	31.69	6	Group 1
G. straminea	93.79	31.95	7	Group 1
G. straminea	92.72	31.75	8	Group 1
G. straminea	92.58	31.77	9	Group 1
G. straminea	91.50	30.60	10	Group 1
G. straminea	97.17	30.46	11	Group 1
G. straminea	96.20	29.65	12	Group 1
G. straminea	100.23	34.63	13	Group 1
G. straminea	100.42	33.30	14	Group 1
G. straminea	100.93	32.65	15	Group 1
G. straminea	100.35	32.30	16	Group 1
G. straminea	101.27	30.82	17	Group 1
G. straminea	100.71	37.06	18	Group 2
G. straminea	101.08	36.43	19	Group 2
G. straminea	99.97	36.65	20	Group 2
G. straminea	97.33	37.45	21	Group 2
G. straminea	97.31	35.42	22	Group 1
G. straminea	102.05	34.00	N/A	N/A
G. straminea	102.11	33.36	N/A	N/A
G. straminea	102.67	34.36	N/A	N/A
G. straminea	101.10	38.41	N/A	N/A
G. straminea	99.50	38.64	N/A	N/A
G. straminea	96.75	29.50	N/A	N/A
G. straminea	102.62	32.00	N/A	N/A
G. straminea	101.78	30.34	N/A	N/A
G. straminea	102.82	31.67	N/A	N/A
G. straminea	102.74	31.73	N/A	N/A
G. straminea	102.48	33.40	N/A	N/A
G. straminea	102.83	37.15	N/A	N/A
G. straminea	100.42	38.48	N/A	N/A
G. straminea	104.00	35.80	N/A	N/A
G. straminea	101.10	36.44	N/A	N/A
G. straminea	100.42	37.22	N/A	N/A
G. straminea	102.65	34.01	N/A	N/A
G. straminea	103.08	37.13	N/A	N/A
G. straminea	96.52	32.07	N/A	N/A
G. straminea	98.37	32.57	N/A	N/A
G. straminea	97.07	31.20	N/A	N/A

G. straminea	100.69	31.50	N/A	N/A
G. straminea	102.95	33.57	N/A	N/A
G. straminea	102.14	34.47	N/A	N/A
G. straminea	101.69	35.05	N/A	N/A
G. straminea	102.47	32.32	N/A	N/A
G. straminea	96.61	32.65	N/A	N/A
G. straminea	98.83	37.35	N/A	N/A
G. straminea	100.10	37.36	N/A	N/A
G. straminea	95.62	32.77	N/A	N/A
G. straminea	100.46	34.54	N/A	N/A
G. straminea	99.64	33.25	N/A	N/A
G. straminea	100.33	38.10	N/A	N/A
G. straminea	101.57	36.23	N/A	N/A
G. straminea	101.37	38.39	N/A	N/A
G. straminea	100.69	34.72	N/A	N/A
G. straminea	101.74	37.02	N/A	N/A
G. straminea	101.62	37.17	N/A	N/A
G. straminea	101.92	35.28	N/A	N/A
G. straminea	91.00	29.62	N/A	N/A
G. straminea	92.35	29.02	N/A	N/A
G. straminea	101.10	36.44	N/A	N/A
G. straminea	100.25	36.57	N/A	N/A
G. straminea	98.29	30.86	N/A	N/A
G. straminea	95.59	31.35	N/A	N/A
G. straminea	95.81	34.12	N/A	N/A
G. straminea	97.92	33.13	N/A	N/A
G. straminea	101.91	36.36	N/A	N/A
G. straminea	100.66	33.27	N/A	N/A
G. straminea	101.22	31.89	N/A	N/A
G. straminea	99.90	35.83	N/A	N/A
G. straminea	102.28	31.85	N/A	N/A
G. straminea	102.56	34.56	N/A	N/A
G. siphonantha	96.15	34.10	23	Group 2
G. siphonantha	100.52	38.14	24	Group 3
G. siphonantha	101.22	36.72	25	Group 2
G. siphonantha	101.08	36.43	26	Group 2
G. siphonantha	98.87	37.10	27	Group 2
G. siphonantha	97.33	37.45	28	Group 2
G. siphonantha	99.88	37.15	29	Group 2
G. siphonantha	101.08	35.05	30	Group 2
G. siphonantha	97.31	35.42	31	Group 2
G. siphonantha	101.78	36.62	32	Group 3
G. siphonantha	102.60	37.44	33	Group 3
G. siphonantha	99.62	38.86	34	Group 3
G. siphonantha	97.66	39.83	35	Group 2
G. siphonantha	94.95	34.85	N/A	N/A
G. siphonantha	97.92	39.61	N/A	N/A
G. siphonantha	97.66	39.58	N/A	N/A
G. siphonantha	98.08	39.57	N/A	N/A
G. siphonantha	98.22	39.49	N/A	N/A
G. siphonantha	97.99	39.39	N/A	N/A

G. siphonantha	99.48	38.55	N/A	N/A
G. siphonantha	101.27	38.01	N/A	N/A
G. siphonantha	100.84	37.98	N/A	N/A
G. siphonantha	101.18	37.79	N/A	N/A
G. siphonantha	101.23	37.75	N/A	N/A
G. siphonantha	100.71	37.69	N/A	N/A
G. siphonantha	100.84	37.68	N/A	N/A
G. siphonantha	101.10	37.66	N/A	N/A
G. siphonantha	101.36	37.50	N/A	N/A
G. siphonantha	97.31	37.47	N/A	N/A
G. siphonantha	102.42	37.39	N/A	N/A
G. siphonantha	101.42	37.37	N/A	N/A
G. siphonantha	101.60	37.30	N/A	N/A
G. siphonantha	98.92	37.21	N/A	N/A
G. siphonantha	99.09	37.19	N/A	N/A
G. siphonantha	101.56	37.18	N/A	N/A
G. siphonantha	101.46	37.18	N/A	N/A
G. siphonantha	102.70	37.17	N/A	N/A
G. siphonantha	98.65	37.06	N/A	N/A
G. siphonantha	102.49	36.94	N/A	N/A
G. siphonantha	101.70	36.93	N/A	N/A
G. siphonantha	102.34	36.89	N/A	N/A
G. siphonantha	102.35	36.77	N/A	N/A
G. siphonantha	102.34	36.67	N/A	N/A
G. siphonantha	101.91	36.35	N/A	N/A
G. siphonantha	101.53	36.31	N/A	N/A
G. siphonantha	94.14	35.73	N/A	N/A
G. siphonantha	94.30	35.70	N/A	N/A
G. siphonantha	102.70	35.59	N/A	N/A
G. siphonantha	102.22	35.30	N/A	N/A
G. siphonantha	99.66	35.20	N/A	N/A
G. siphonantha	98.85	35.13	N/A	N/A
G. siphonantha	101.51	35.08	N/A	N/A
G. siphonantha	101.82	35.07	N/A	N/A
G. siphonantha	101.44	34.86	N/A	N/A
G. siphonantha	101.54	34.85	N/A	N/A
G. siphonantha	102.26	34.74	N/A	N/A
G. siphonantha	98.06	34.68	N/A	N/A
G. siphonantha	99.73	34.65	N/A	N/A
G. siphonantha	95.31	34.58	N/A	N/A
G. siphonantha	100.55	34.55	N/A	N/A
G. siphonantha	100.55	34.55	N/A	N/A
G. siphonantha	99.19	34.48	N/A	N/A
G. siphonantha	94.63	34.48	N/A	N/A
G. siphonantha	100.21	34.36	N/A	N/A
G. siphonantha	95.75	34.16	N/A	N/A
